# Genome Streamlining, Plasticity, and Metabolic Versatility Distinguish Co-occurring Toxic and Nontoxic Cyanobacterial Strains of *Microcoleus*

**DOI:** 10.1128/mBio.02235-21

**Published:** 2021-10-26

**Authors:** Hwee Sze Tee, Susanna A. Wood, Keith Bouma-Gregson, Gavin Lear, Kim M. Handley

**Affiliations:** a School of Biological Sciences, The University of Auckland, Auckland, New Zealand; b Cawthron Institute, Nelson, New Zealand; c U.S. Geological Survey, California Water Science Center, Sacramento, California, USA; CEH-Oxford

**Keywords:** *Phormidium autumnale*, anatoxins, benthic cyanobacteria, comparative genomics, cyanobacterial proliferation, resource trade-off, toxic and nontoxic cyanobacteria

## Abstract

Harmful cyanobacterial bloom occurrences have increased worldwide due to climate change and eutrophication, causing nuisance and animal deaths. Species from the benthic cyanobacterial genus *Microcoleus* are ubiquitous and form thick mats in freshwater systems, such as rivers, that are sometimes toxic due to the production of potent neurotoxins (anatoxins). Anatoxin-producing (toxic) strains typically coexist with non-anatoxin-producing (nontoxic) strains in mats, although the reason for this is unclear. To determine the genetic mechanisms differentiating toxic and nontoxic *Microcoleus*, we sequenced and assembled genomes from 11 cultures and compared these to another 31 *Microcoleus* genomes. Average nucleotide identities (ANI) indicate that toxic and nontoxic strains are distinct species (ANI, <95%), and only 6% of genes are shared across all 42 genomes, suggesting a high level of genetic divergence among *Microcoleus* strains. Comparative genomics showed substantial genome streamlining in toxic strains and a potential dependency on external sources for thiamine and sucrose. Toxic and nontoxic strains are further differentiated by an additional set of putative nitrate transporter (nitrogen uptake) and cyanophycin (carbon and nitrogen storage) genes, respectively. These genes likely confer distinct competitive advantages based on nutrient availability and suggest nontoxic strains are more robust to nutrient fluctuations. Nontoxic strains also possess twice as many transposable elements, potentially facilitating greater genetic adaptation to environmental changes. Our results offer insights into the divergent evolution of *Microcoleus* strains and the potential for cooperative and competitive interactions that contribute to the co-occurrence of toxic and nontoxic species within mats.

## INTRODUCTION

Cyanobacteria first appeared on Earth over two billion years ago and are credited with the evolution of aerobic life (1). Despite this contribution, the increased occurrence of cyanobacterial planktonic blooms and benthic proliferations, due to anthropogenic activity, has severely disrupted aquatic habitats and deteriorated water quality (2). Cyanobacterial blooms and proliferations are often composed of both toxic and nontoxic strains, with various amounts of toxin detected spatially and temporally (3–7). However, the genomic differences that lead to the dominance of either toxic or nontoxic strains is unknown.

Here, we focus on the benthic cyanobacterial genus *Microcoleus*, which inhabits freshwater systems worldwide (8–10). Under favorable environmental and hydrological conditions, *Microcoleus* forms cohesive mats, which can cover large areas in lakes and rivers (11). Some *Microcoleus* species produce a neurotoxin (anatoxin-a) that has been linked to animal deaths in many countries, including the United States, the Netherlands, New Zealand, Germany, and France (12), and can accumulate in aquatic organisms (13). Both anatoxin-producing and non-anatoxin-producing *Microcoleus* strains (henceforth referred to as toxic and nontoxic strains, respectively) are often found co-occurring within a single mat (e.g., coating a riverbed cobble), where their relative abundances determine the toxin concentration in the mat (5, 11, 14, 15). Although benthic proliferations of only nontoxic strains have been documented (11), proliferations containing toxic strains are always reported as a mixture of toxic and nontoxic strains (16).

Unlike planktonic cyanobacteria, which often bloom at high nutrient concentrations (17, 18), *Microcoleus* proliferates under low-phosphate and slightly elevated nitrogen conditions by adopting diverse strategies to scavenge phosphorus and nitrogen (8, 19). Toxic *Microcoleus* strains have been reported to occur at higher nitrogen levels than nontoxic strains (8), although cellular anatoxin concentrations have been experimentally shown to be lowest under high-nitrogen and high-phosphorus conditions (14), as reported for several other cyanobacteria and cyanotoxins (20). In contrast, nontoxic *Microcoleus* strains are reported to have higher cell concentrations and higher maximum growth rates than toxic strains, regardless of nutrient concentrations (phosphorus and nitrogen), suggesting higher energy costs associated with toxin production (14).

Comparative genomics has highlighted marked genotypic and phenotypic plasticity within cyanobacterial species, such as planktonic Raphidiopsis raciborskii and Microcystis aeruginosa, and benthic and planktonic *Planktothrix* spp. (6, 21–25). Striking differences in genes found between toxic and nontoxic *R. raciborskii* strains suggest that toxin production may be associated with stress response (26). However, pangenomic comparisons differentiating other toxic and nontoxic cyanobacterial species are in general lacking, and none exist for benthic species or anatoxin producers (27, 28). As such, the genetic mechanisms that regulate the ecological successes of toxic and nontoxic benthic cyanobacteria, such as *Microcoleus*, remain elusive.

We investigated the genetic differences between toxic and nontoxic *Microcoleus* strains to determine the relationship between these often cohabiting and closely related benthic cyanobacterial species. For this, we compared 42 *Microcoleus* metagenome-assembled genomes (MAGs), including 30 nontoxic and 12 toxic strains, sourced from enrichment cultures or *Microcoleus*-dominated mats from different rivers in New Zealand and the United States. We hypothesized that there would be marked differences in the genomes of toxic and nontoxic strains, linked to their different growth rates associated with N availability. Our results help to explain why toxic strains never proliferate in the absence of nontoxic strains and the association between toxic strains and higher N availability.

## RESULTS AND DISCUSSION

### Genome reduction among toxic *Microcoleus* strains.

To compare the genomes of toxic and nontoxic *Microcoleus*, we first sequenced and assembled the genomes (metagenome-assembled genomes, MAGs) of 11 nonaxenic isolates (3 toxic and 8 nontoxic) related to *Microcoleus autumnalis* (previously Phormidium autumnale) (29). Production of anatoxin-a, homoanatoxin-a, or chemical variants of these was confirmed for the 3 isolates with anatoxin gene clusters by liquid chromatography coupled with tandem mass spectrometry (LC-MS/MS) (Table S1). We then compared these to a further 31 MAGs previously obtained from nonaxenic isolates (3 toxic) and mats sampled from rivers (6 toxic, 22 nontoxic) (8, 19, 30), where the 6 mat-derived MAGs with anatoxin gene clusters were spatially associated with anatoxins (8, 19). The 42 *M. autumnalis*-like genomes were classified based on the presence/absence of the anatoxin gene cluster. Each had an estimated completeness of >80% (contamination scores of 0.7 ± 1.3%; Table S1).

10.1128/mBio.02235-21.5 TABLE S1 *Microcoleus* strain and reference descriptions*^a^*
*^a^*Strains/references with anatoxin gene cluster are shaded with a gray background. Anatoxin-a is detected via LC-MS/MS or anatoxin gene cluster is present in the genome (+); no anatoxin-a is detected or no anatoxin gene cluster is found in the genome (–); samples were not tested for anatoxin-a (nt). Download Table S1, XLSX file, 0.02 MB. Copyright © 2021 Tee et al. 2021 Tee et al. https://creativecommons.org/licenses/by/4.0/ This content is distributed under the terms of the Creative Commons Attribution 4.0 International license.

**FIG 1:**
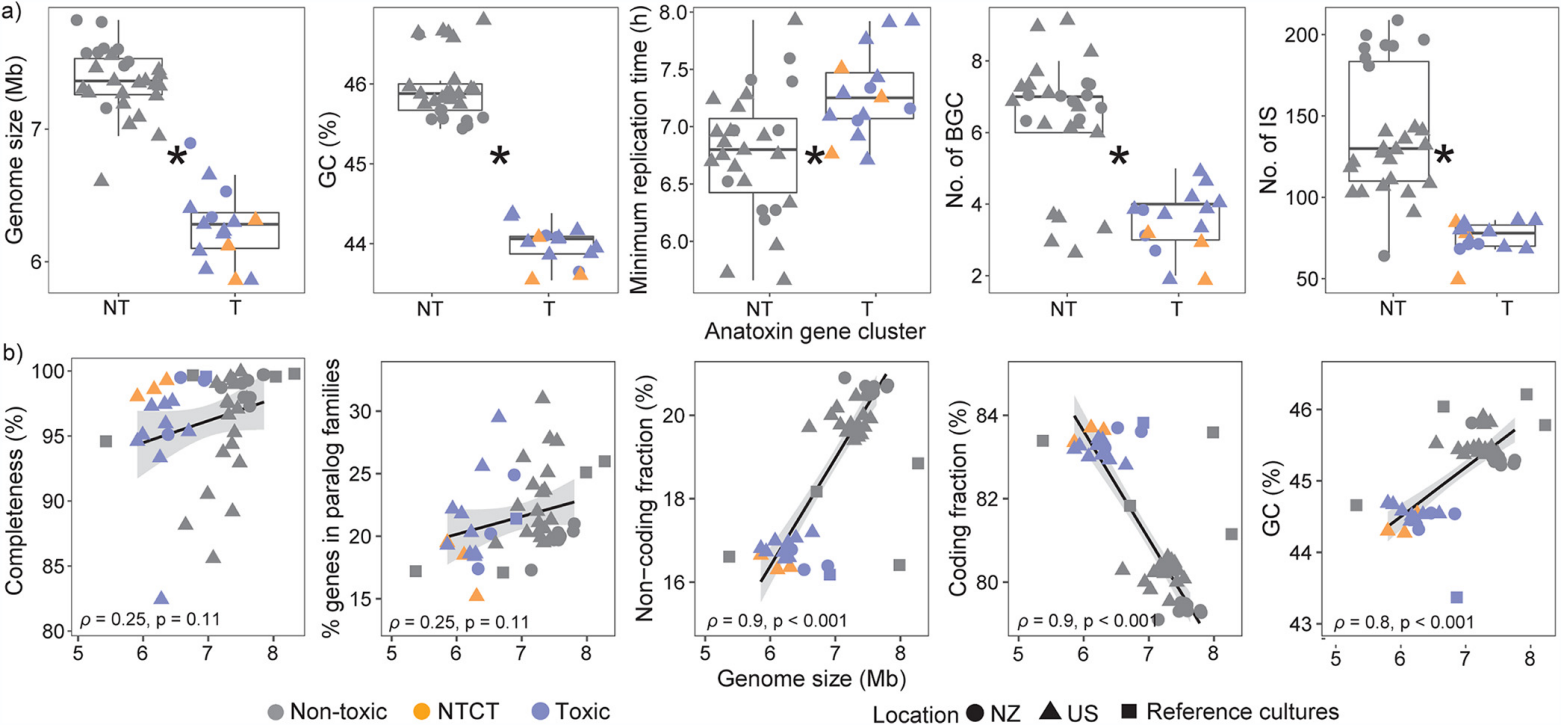
Genomic differences between nontoxic and toxic *Microcoleus* strains. The symbol color indicates genomes that are nontoxic (NT; gray), nontoxic clustered with toxic (NTCT; orange), and toxic (T; blue), and the shape represents the reference culture (square) or *Microcoleus* strains that originated from New Zealand (NZ; circle) and the United States (US). (a) Boxplots of nontoxic and toxic *Microcoleus* genomes showing estimated genome sizes, number of biosynthetic gene clusters (BGC), number of insertion sequences (IS), percent GC content, and predicted minimum replication time. Boxes represent the interquartile range (IQR) between the first and third quartiles, and the horizontal line inside the box represents the median. Whiskers represent the lowest and highest values within 1.5 times the IQR from the first and third quartiles, respectively. The asterisk (∗) indicates a significant difference between nontoxic and toxic *Microcoleus* genomes. (b) Correlations between genome size and the coding/noncoding fraction, GC content, percentage of genes in paralog families, and genome completeness. Regression lines, Pearson correlation coefficients (ρ), and the associated *P* values are shown in the plots.

Differences in bacterial genome sizes typically reflect gain or loss of function and adaptation to more defined niches in smaller streamlined genomes (31, 32). Results show the toxic *Microcoleus* have significantly smaller estimated genome sizes (∼6.3 ± 0.3 Mbp) and lower GC contents (∼44.5 ± 0.1%) than the nontoxic ones, which have estimated genome sizes of ∼7.4 ± 0.3 Mbp and ∼45.5 ± 0.2% GC content (Fig. 1a). This excludes three nontoxic *Microcoleus*, which have genome sizes and GC contents comparable to those of toxic *Microcoleus* and features suggestive of recent toxic gene cluster loss (discussed below). They were therefore analyzed separately (and are defined here as nontoxic clustered with toxic strains, NTCT). As most genomes of smaller toxic strains were >90% complete, their smaller size is not expected to reflect assembly or binning errors (Fig. 1b). Confirming this, we compared the genome size and assembly completeness of all toxic and nontoxic strains and observed a poor correlation (*ρ* = 0.25).

While the chemistry and biosynthesis of cyanotoxins are highly diverse, and they may function differently (20, 33), experimental and field-based evidence indicates that, like *Microcoleus*, other toxic and nontoxic cyanobacteria also differ in their requirements for nutrients (8, 34–37). We therefore sought to determine whether the observed difference in genome size in *Microcoleus* is also found in other cyanobacterial taxa. While we observed significantly different genome sizes between toxic and nontoxic strains of Microcystis aeruginosa (*n* = 25, *P* value < 0.05), no correlation was found between genome size and toxin production of other toxin-producing cyanobacteria (i.e., *Anabaena*, *Dolichospermum*, and *Raphidiopsis raciborskii*; *P* value > 0.05) (Table S2). This implies that the genomic structures of *Microcoleus* spp. and Microcystis aeruginosa are highly distinct from other cyanobacteria. The limited number of genomes available for the other cyanobacteria (*n* < 10) may also lead to nonsignificant results, suggesting more comprehensive sequencing is needed to analyze their genome size distributions.

10.1128/mBio.02235-21.6 TABLE S2 Genome size of other reference strains*^a^*
*^a^*Strains with toxin gene clusters detected are shaded with a gray background. Download Table S2, XLSX file, 0.01 MB. Copyright © 2021 Tee et al. 2021 Tee et al. https://creativecommons.org/licenses/by/4.0/ This content is distributed under the terms of the Creative Commons Attribution 4.0 International license.

Significant differences in genome sizes between toxic and nontoxic *Microcoleus* strains may contribute to variations in genome plasticity and ecological adaptation (31). No association was found between genome size and the fraction of paralogous genes (Fig. 1b), a feature of genome streamlining (38). However, the non-protein-coding fractions were positively correlated with genome size and lower in toxic strains (16.8 ± 0.3%) than in nontoxic strains (20.1 ± 0.5%). This suggests that toxic strains may have undergone genome streamlining, retaining fewer genes involved in transcriptional and translational regulation (32). Some bacterial lineages streamline their genomes to select against cell complexity and reduce replication cost (31, 32). Previous research has shown that anatoxin-producing strains have lower cell concentrations and growth rates under culture conditions (14). Our results illustrate that toxic *Microcoleus* strains have significantly longer predicted minimum replication times of ∼7.3 ± 0.4 h, compared to nontoxic strains (∼6.8 ± 0.5 h; Fig. 1a), indicating a potential trade-off between toxin production and growth. In contrast, because nontoxic strains have larger genome sizes, they may harbor enhanced metabolic capabilities that promote proliferation.

### Phylogenetic and pangenome analyses demonstrate toxic and nontoxic *Microcoleus* strains comprise distinct groups of species.

**FIG 2:**
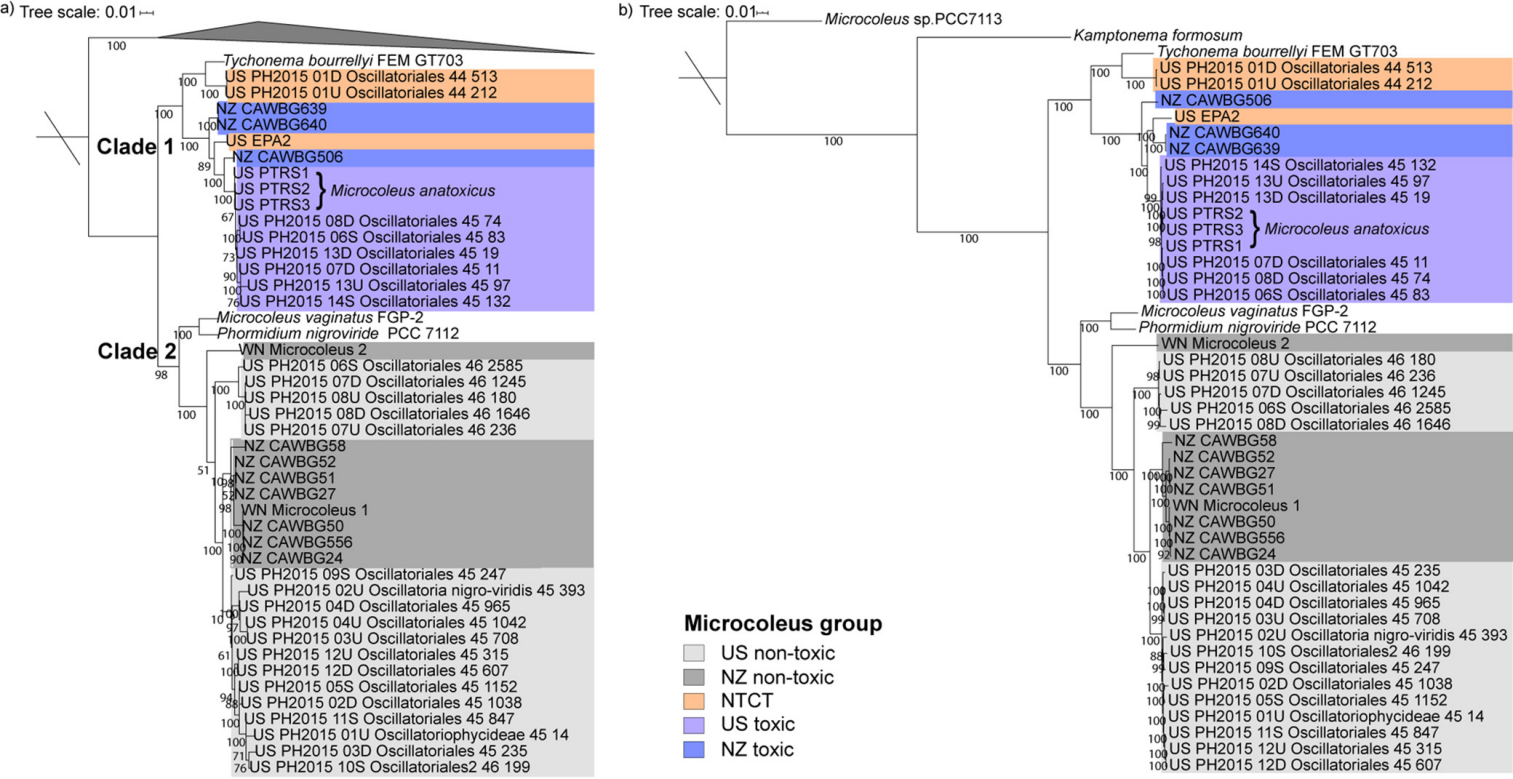
Maximum likelihood trees of the single-copy genes present in *Microcoleus* spp. (a) Phylogenetic tree of concatenated alignments of 120 core single-copy marker genes. The tree is rooted with 12 other cyanobacterial references as shown in Table S1. (b) Species tree based on 525 core single-copy orthologs present in all *Microcoleus* genomes. The tree is rooted with *Microcoleus* sp. strain PCC7113. For both trees, scale bars represent the number of substitutions per site. Bootstrap values over 50% are shown. Color demarks nontoxic (non-anatoxin-producing), and toxic (anatoxin-producing) *Microcoleus* groups from the United States (US) and New Zealand (NZ). US PTRS1, 2, and 3 are known as Microcoleus anatoxicus in Conklin et al. (30). Oscillatoria nigro-viridis (in NCBI, UniProt) is known as Phormidium nigroviride in AlgaeBase (https://www.algaebase.org/) and *Microcoleus* sp. in Genome Taxonomy Database (https://gtdb.ecogenomic.org/). NTCT, nontoxic clustered with toxic.

A phylogenetic tree of cyanobacterial 16S rRNA genes showed that all *Microcoleus* strains were placed within a highly supported cluster (98% bootstrap value) with *Tychonema* CCAP, Microcoleus vaginatus, Phormidium nigroviride, and Phormidium autumnale (Fig. S1), in line with previous findings (8, 29). As 16S rRNA analysis can typically only resolve to the genus level (39, 40), we generated maximum-likelihood phylogenies from alignments of 120 core single-copy marker genes and 525 core single-copy orthologs present in all genomes (Fig. 2), as well as an alignment of 12,406 single-nucleotide polymorphisms (Fig. S2). All trees revealed two distinct *Microcoleus* clades, comprising either toxic (and NTCT) or nontoxic species (clades 1 and 2, respectively) (Fig. 2). Both toxic and nontoxic strains were further divided broadly based on geography. Average nucleotide identity (ANI) and digital DNA-DNA hybridization (dDDH) analyses (Fig. S3) predict that the 42 *Microcoleus* genomes derived from 9 species (thresholds for ANI, 96.5%; for dDDH, 70%) (41, 42). These comprise three closely related anatoxin-producing species (clusters 4, 5, and 7), two NTCT species (clusters 6 and 8) (Fig. S3), and four non-anatoxin-producing species.

10.1128/mBio.02235-21.1 TEXT S1 Additional gene clusters related to chemosensory and stress adaptation in nontoxic *Microcoleus*. Download TEXT S1, DOCX file, 0.02 MB. Copyright © 2021 Tee et al. 2021 Tee et al. https://creativecommons.org/licenses/by/4.0/ This content is distributed under the terms of the Creative Commons Attribution 4.0 International license.

10.1128/mBio.02235-21.1 FIG S1 Maximum likelihood trees of the predicted full-length cyanobacterial 16S ribosomal RNA genes. Scale represents the number of substitutions per site, and bootstrap values over 50% are shown. The box color indicates the nontoxic (non-anatoxin-producing) and toxic (anatoxin-producing) *Microcoleus* group from the United States (US) and New Zealand (NZ). The tree is rooted with Pseudanabaena tremula UTCC 471. NTCT, nontoxic clustered with toxic. Note that *Oscillatoria nigro-viridis* (in NCBI, UniProt) is known as *Phormidium nigroviride* in AlgaeBase (https://www.algaebase.org/) and *Microcoleus* sp. in Genome Taxonomy Database (https://gtdb.ecogenomic.org/). Download FIG S1, PDF file, 1.6 MB. Copyright © 2021 Tee et al. 2021 Tee et al. https://creativecommons.org/licenses/by/4.0/ This content is distributed under the terms of the Creative Commons Attribution 4.0 International license.

10.1128/mBio.02235-21.2 FIG S2 Maximum likelihood trees of the core single-nucleotide polymorphism. Phylogenetic tree of 12,406 core single-nucleotide polymorphisms identified among study strains. Scale represents the number of substitutions per site, and bootstrap values over 50% are shown. The box color indicates the nontoxic (non-anatoxin-producing) and toxic (anatoxin-producing) *Microcoleus* group from the United States (US) and New Zealand (NZ). Other names for *Oscillatoria nigro-viridis* (NCBI, UniProt) are *Phormidium nigroviride* (AlgaeBase) and *Microcoleus* sp. (GTDB). The tree is rooted with *Microcoleus* sp. PCC7113. NTCT, nontoxic clustered with toxic. Download FIG S2, EPS file, 0.8 MB. Copyright © 2021 Tee et al. 2021 Tee et al. https://creativecommons.org/licenses/by/4.0/ This content is distributed under the terms of the Creative Commons Attribution 4.0 International license.

10.1128/mBio.02235-21.3 FIG S3 Genome relatedness of the *Microcoleus* species in our data set, along with 15 other cyanobacterial species retrieved from the NCBI genome database (Table S1). The heatmap shows the pairwise percentage average nucleotide identity (ANI). Hierarchical clustering is based on the ward.D2 algorithm. The plot color gradient (blue-yellow-red) indicates the percentage similarity. Clusters (or sole genomes) representing presumed distinct *Microcoleus* species are numbered 1 to 9 (dark red squares). The text color indicates reference genomes (black) and *Microcoleus* that are nontoxic (grey), nontoxic clustered with toxic (orange), and toxic (blue). Download FIG S3, PDF file, 2.8 MB. Copyright © 2021 Tee et al. 2021 Tee et al. https://creativecommons.org/licenses/by/4.0/ This content is distributed under the terms of the Creative Commons Attribution 4.0 International license.

*Microcoleus* strains vary in their cell and filament dimensions, coloration, and morphology of their apical cells (i.e., obtusely rounded or pointed or with/without calyptra); there is no consistency with these morphological features and the phylogeny presented (3, 5). Based on our observations, we also note that these features can vary over time in culture and that strains in culture often do not have the exact same morphological features that were present when they were in the environment. Therefore, it is crucial to incorporate molecular characterization for species/strain identification.

**FIG 3:**
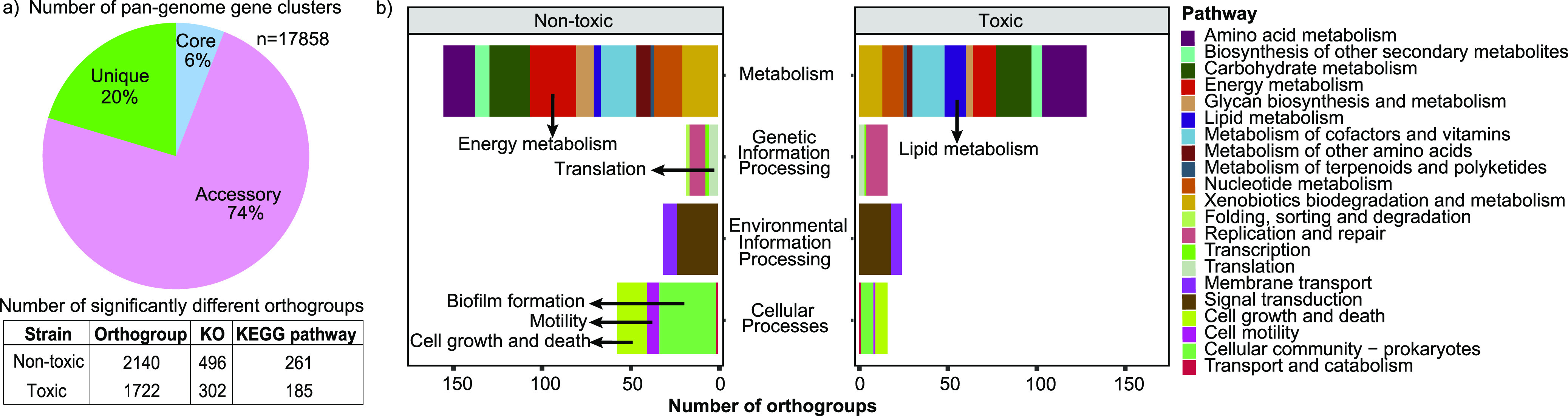
Pan-genome gene clusters and the orthogroups that were significantly higher in nontoxic or toxic groups. (a) The pie chart indicates the percentage of orthogroups that were classified as unique, accessory, or core according to their distribution among genomes. The orthogroups were annotated with KEGG Orthology (KO) and mapped to the KEGG pathway database. (b) Bar plots indicate the sum of the number of orthogroups that were significantly higher in either nontoxic or toxic strains. The orthogroups were categorized according to their KEGG functional pathway.

Pangenome analysis identified a total of 17,858 gene clusters (Markov cluster algorithm [MCL] inflation = 1.5) among 42 *Microcoleus* strains and 5 cyanobacterial reference genomes. Only 6% of gene clusters were present in all genomes, and 18% were unique, suggesting huge genetic variability among *Microcoleus* strains (Fig. 3 and Table S3). Specialist cyanobacterial *Raphidiopsis* spp. share a highly conserved core genome (2,125 out of 4,715 orthologous gene clusters), while generalist *Microcystis* spp. exhibit considerable genetic divergence (413/13,884 orthologous gene clusters) (28). This suggests *Microcoleus* spp., which have relatively large genomes and high genomic diversity, may adopt a generalist approach to adapt to broad ranges of environments (43). A total of 2,140 and 1,722 orthogroups/gene clusters were significantly more prevalent in nontoxic and toxic strains, respectively (Benjamini-Hochberg adjusted *P* value < 0.05; Fig. 3 and Table S4), highlighting large differences in gene content and resource allocation between toxic and nontoxic strains that may contribute to distinct physiological responses toward environmental disturbances.

10.1128/mBio.02235-21.7 TABLE S3 Orthogroups identified per genome using OrthoFinder v2.3.3 with default parameters. Download Table S3, XLSX file, 2.7 MB. Copyright © 2021 Tee et al. 2021 Tee et al. https://creativecommons.org/licenses/by/4.0/ This content is distributed under the terms of the Creative Commons Attribution 4.0 International license.

10.1128/mBio.02235-21.8 TABLE S4 Orthogroups that are significantly prevalent in toxic/nontoxic strains. Download Table S4, XLSX file, 0.2 MB. Copyright © 2021 Tee et al. 2021 Tee et al. https://creativecommons.org/licenses/by/4.0/ This content is distributed under the terms of the Creative Commons Attribution 4.0 International license.

### Evidence of anatoxin gene cluster loss and potential to disrupt the DNA phosphorothioation stress response mechanism.

**FIG 4:**
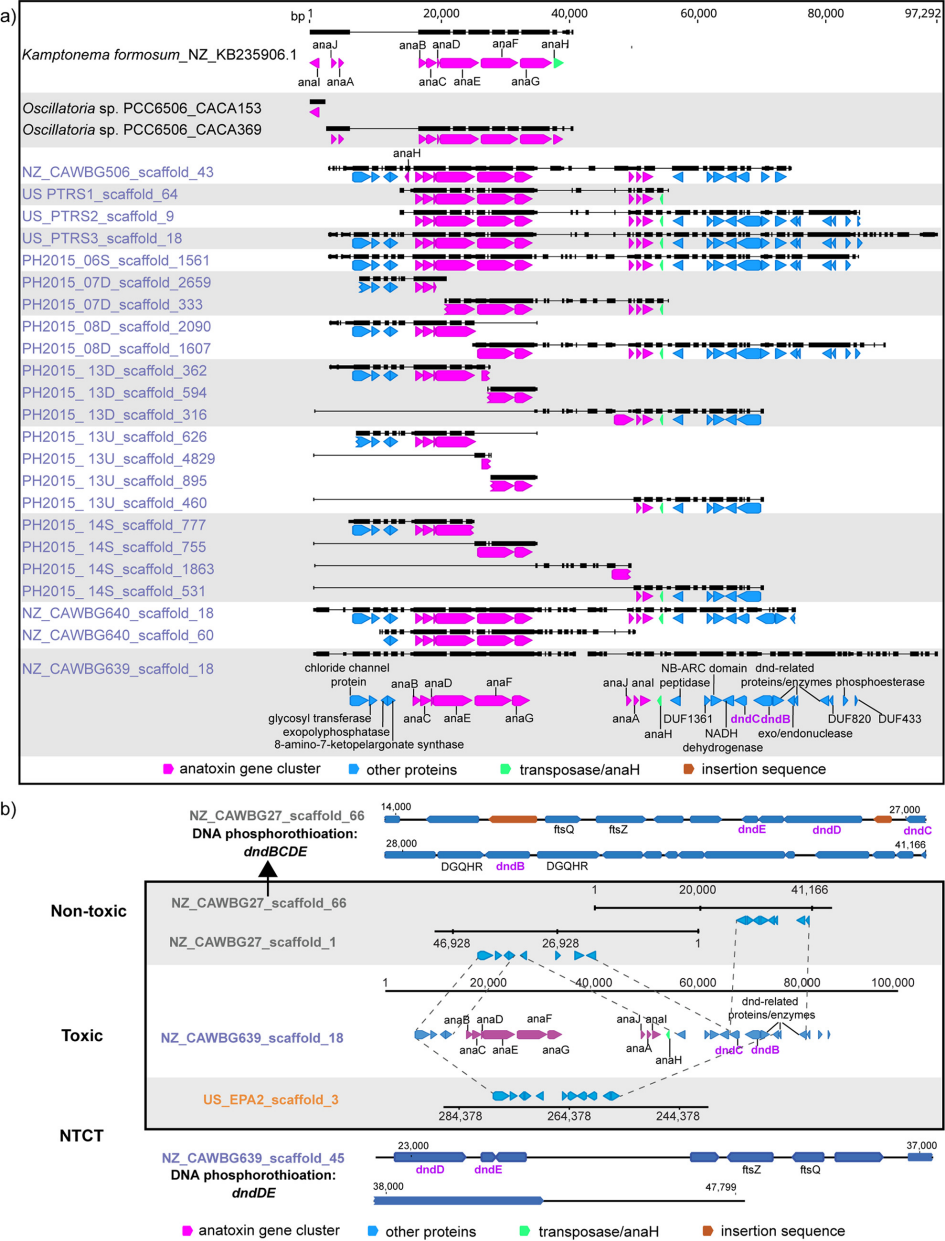
Multiple sequence alignments of anatoxin gene clusters and the neighboring genes. Black horizontal lines represent single contigs. Genes related to DNA phosphorothioation (*dndBCDE*) are indicated in bold purple font. Alternating white-gray shading denotes contigs from the same genome/MAG. (a) Comparison of anatoxin gene clusters present in *Microcoleus* genomes (blue font). Anatoxin gene clusters from *Kamptonema formosum* and *Oscillatoria* sp. PCC6506 (black font) are used as references. (b) Comparison of genes neighboring the anatoxin gene cluster in *Microcoleus* genomes. The dotted lines represent the matching sections between toxic (unshaded box) and nontoxic strains (shaded box). The DNA phosphorothioation gene cluster, *dndBCDE*, from a representative nontoxic strain is shown at the top and is contained within a single contig. The *dndD* and *dndE* genes from a representative toxic strain are located on different contigs (bottom). NTCT, nontoxic clustered with toxic.

To investigate the genomic structure of the anatoxin gene cluster in *Microcoleus* strains, we aligned the clusters, along with neighboring genes. The *anaI*, *anaJ*, and *anaA* genes in all *Microcoleus* strains were rearranged and located downstream of *anaG*, compared to Kamptonema formosum and *Oscillatoria* sp. strain PCC6506 genomes (Fig. 4a). Transposase (*anaH*) genes were found next to the anatoxin gene clusters, highlighting the possibility of horizontal gene transfer and random gene rearrangement/loss. The order within *Microcoleus* anatoxin gene clusters was highly conserved, suggesting these genes were acquired through vertical gene transfer. One of the *Microcoleus* strains, NZ-CAWBG640, also harbored a second copy of the anatoxin gene cluster (Fig. 4a). These two gene cluster copies were highly similar, with 98% alignment coverage and 99.9% identity, implying either cobinning of a toxin cluster belonging to a conspecific strain (∼3% predicted strain heterogeneity based on CheckM) or a gene duplication event. Potential duplication of toxin clusters would underline the importance of anatoxin production to these organisms (44, 45) and warrants further investigation. While the microcystin gene cluster is generally considered to be single copy (46, 47), a few studies have indicated that certain biosynthetic gene clusters, including nrps/pks (45), bacteriocins (48), and microginin (49), are present in multiple copies in cyanobacterial genomes.

Despite the absence of anatoxin gene clusters in the NTCT strains, genes neighboring the cluster in the toxic strains preserved a similar structural arrangement within the genomes of NTCT strains, including a colocated partial DNA modification/phosphorothioation cluster, *dndBC* (Fig. 4b). Multiple toxin gene loss events are predicted for *Microcystis* strains throughout their evolution, based on the heterogenous phylogeny of toxic and nontoxic strains (50). Evidence here suggests recent loss of the anatoxin cluster from the NTCT *Microcoleus* strains, which is further supported by the high level of ANI they share with their toxic relatives and their comparably small genome sizes (∼6.1 ± 0.2 Mbp), low GC content (44.4 ± 0.1%), and low fraction of noncoding genes (Fig. 1a). Other nontoxic strains were instead equipped with a colocated, but complete, DNA modification cluster, *dndBCDE*, which functions to replace a nonbridging oxygen atom in the phosphodiester bond with sulfur to protect against nuclease activity or oxidative stress (51, 52). The presence of a DNA phosphorothioation system type-III restriction enzyme (RE) upstream of this *dndBCDE* cluster indicates that DNA phosphorothioation in *Microcoleus* likely functions as a restriction-modification (R-M) system, affording protection against phage by destroying unmodified foreign DNA (53).

Both *dndBC* and *dndDE* were present in toxic and NTCT strains but distributed across different contigs (as in toxic *M. aeruginosa* strains [54]) and separated by at least 40 kb (Fig. 4b), possibly influencing the expression and functionality of *dnd* gene products. The anatoxin gene cluster and incomplete *dnd* gene cluster (*dndBC*) are located near a type-III RE. Without a complete *dnd* gene cluster that functions as a DNA phosphorothioation system, the RE itself may induce programmed cell death (PCD) or cell lysis under stressful conditions (55) and subsequent release of the intracellular cyanotoxins into the environment (56). The functional coupling between PCD and microcystin release has been shown to promote the survival of the remaining *Microcystis* population under stress by promoting colony formation (57) and reducing grazing pressure (58). While anatoxins may behave differently than microcystin, we posit that toxic *Microcoleus* strains may undergo PCD and release intracellular anatoxin in response to stress, which likely offers protection to the wider mat community.

### Nontoxic *Microcoleus* strains harbor diverse biosynthetic gene clusters.

**FIG 5:**
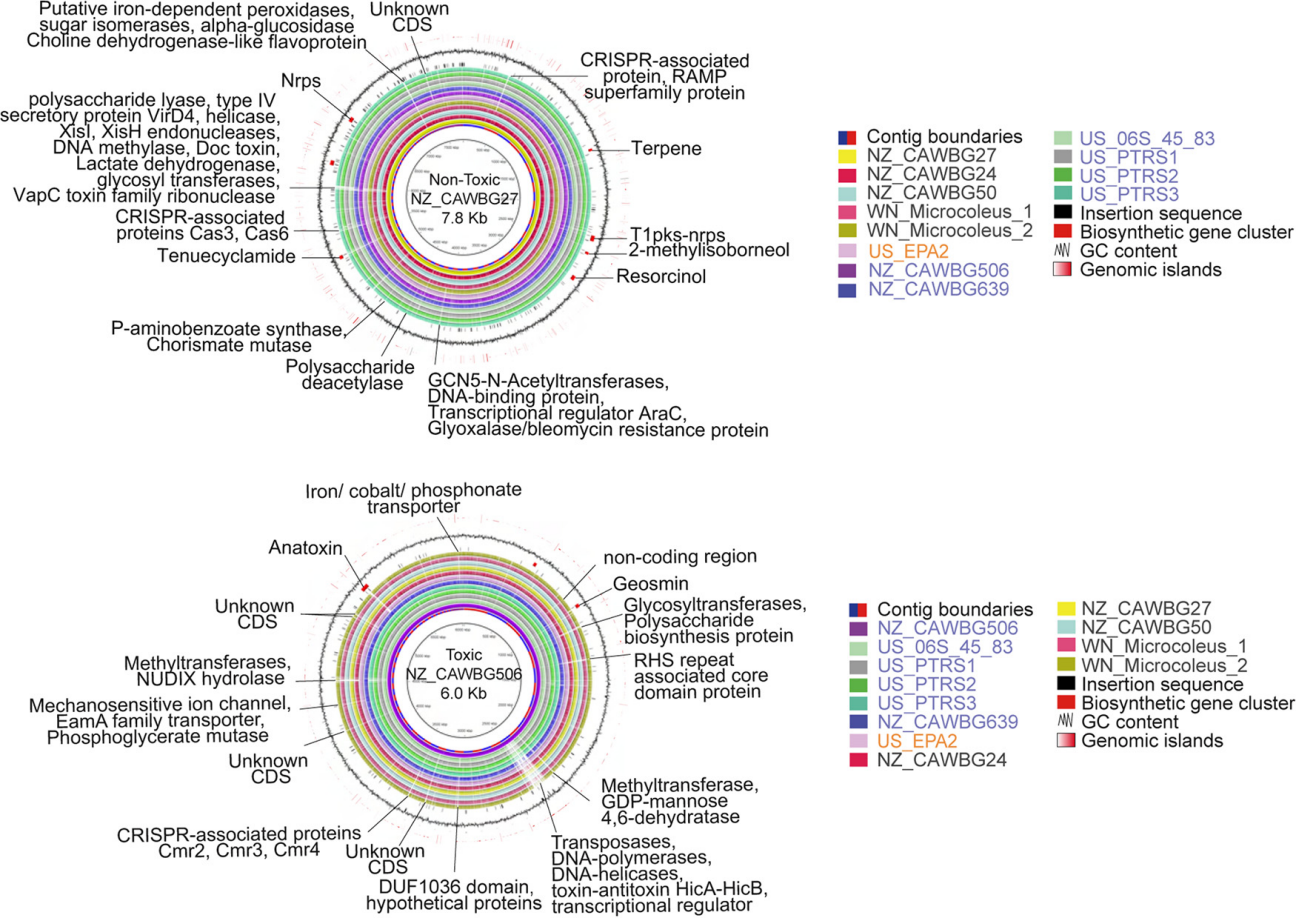
Whole-genome comparison plot. Circular maps of the alignment of *Microcoleus* genomes with a nontoxic (NZ-CAWBG27, top) or toxic (NZ-CAWBG506, bottom) strain as reference, and reference strain contigs ordered by size. The intensity of line color in the circular plot represents BLAST hit identity scores, while blanks indicate no match or nonsignificant matches. The first and second inner rings represent contig boundaries and reference genomes, respectively. From the 3^rd^ to the 13th ring, each ring corresponds to a different genome. The 14th ring represents the insertion sequence, followed by biosynthetic gene clusters and GC content. The outermost ring corresponds to the genomic island score predicted with Alien Hunter v1.7.1. Notable features are labeled (e.g., BGCs and discrepancies between toxic and nontoxic genomes).

In concordance with genome size, toxic *Microcoleus* strains harbor significantly fewer biosynthetic gene clusters (BGCs) and insertion sequences (IS) than their nontoxic counterparts (Fig. 1a). Insertion sequences (IS) were widespread among all *Microcoleus* strains (1 to 3.6% of total genomic DNA; Fig. 5) although disproportionately more numerous in nontoxic strains (Fig. 1a). IS were largely associated with BGC and genomic island occurrences (Fig. 1a and 5) and likely facilitate genetic variation. BGCs are common in cyanobacteria and are responsible for the production of secondary metabolites, including toxins, antibiotics, and siderophores (45, 59). The production of secondary metabolites or cyanotoxins is closely linked to cyanobacterial evolution (2, 60) and may offer cyanobacteria a competitive or physiological advantage, allowing them to adapt and survive under a greater range of conditions (45, 61, 62). For example, secondary metabolites may offer protection against grazing/predation, UV radiation, and oxidative stress or promote efficient acquisition of limited nutrients (2, 60, 61).

**FIG 6:**
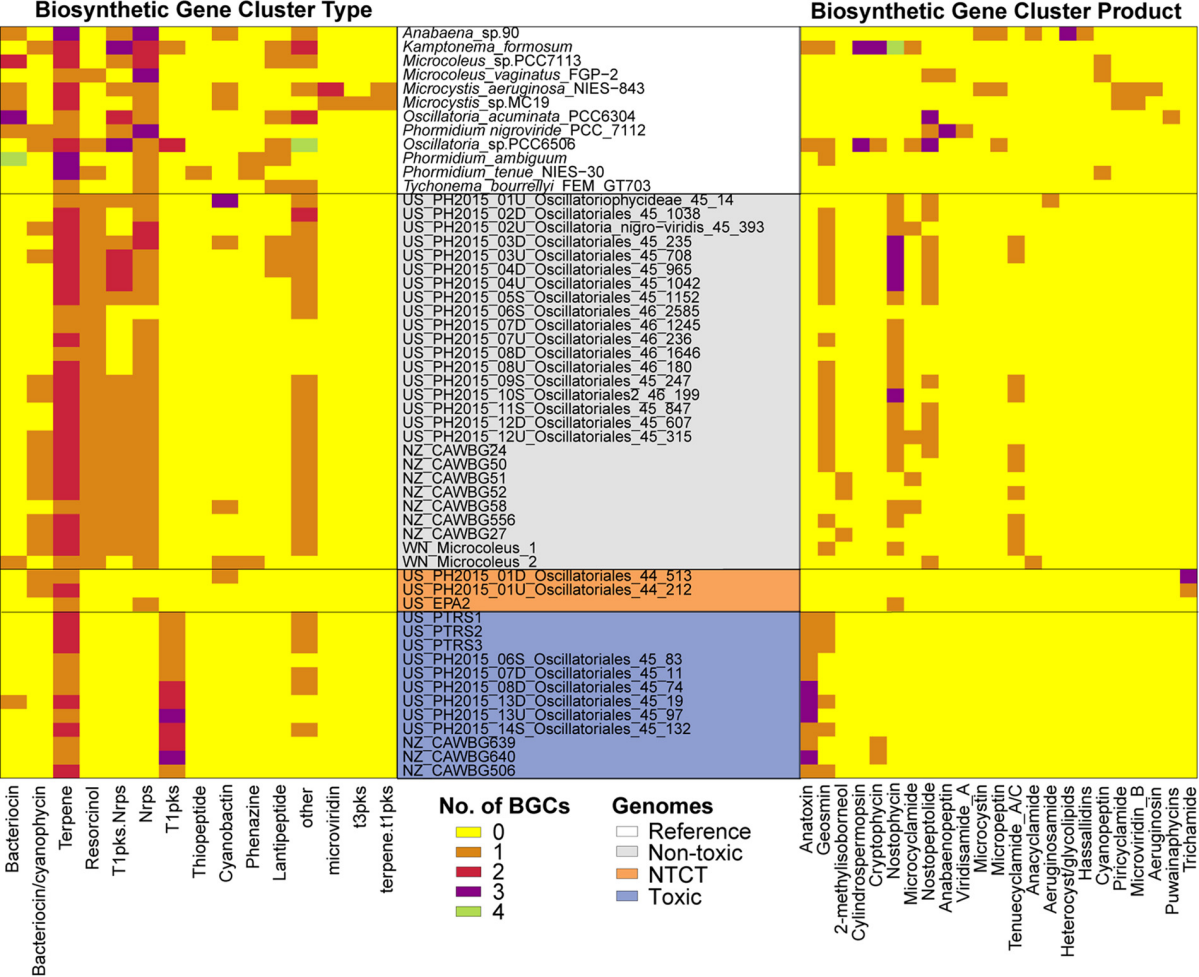
Heatmaps of cyanobacterial biosynthetic gene clusters (BGCs) identified among the *Microcoleus* spp. Heatmaps show the type of BGCs (left) and the known class of BGCs (right) identified among the genomes. The background color for text indicates reference genomes (black) and *Microcoleus* strains that are nontoxic (gray), nontoxic clustered with toxic (NTCT; orange), and toxic (blue). The data in the heatmaps were assigned individual colors based on the number of clusters found.

Although toxic *Microcoleus* have few BGCs, besides the t1pks that is encoded by the ∼29-kb anatoxin gene cluster (Fig. 5 and 6), geosmin and 2-methylisoborneol genes were detected in some toxic and nontoxic strains. These cause an earthy/musty odor in aquatic systems and are a nuisance to drinking water systems (63), which results indicate is a feature of both *Microcoleus* groups. However, we found nonribosomal peptide-synthetase (nrps), bacteriocin, resorcinol, and a type-I polyketide synthase (t1pks)-nrps hybrid BGCs only in nontoxic *Microcoleus* (Fig. 6). These types of BGCs are used to produce antibacterial toxin compounds, including tenuecyclamide, nostopeptolide, and nostophycin (Fig. 6), suggesting that non-anatoxin-producing *Microcoleus* can produce a suite of antibacterial compounds and may exhibit cytotoxic activity (62). Overall, results indicate that anatoxin gene loss (or lack of acquisition) may be offset by an increase in BGCs, which could drive adaptive diversification among nontoxic *Microcoleus* strains.

### Large metabolic differences between toxic and nontoxic groups.

**FIG 7:**
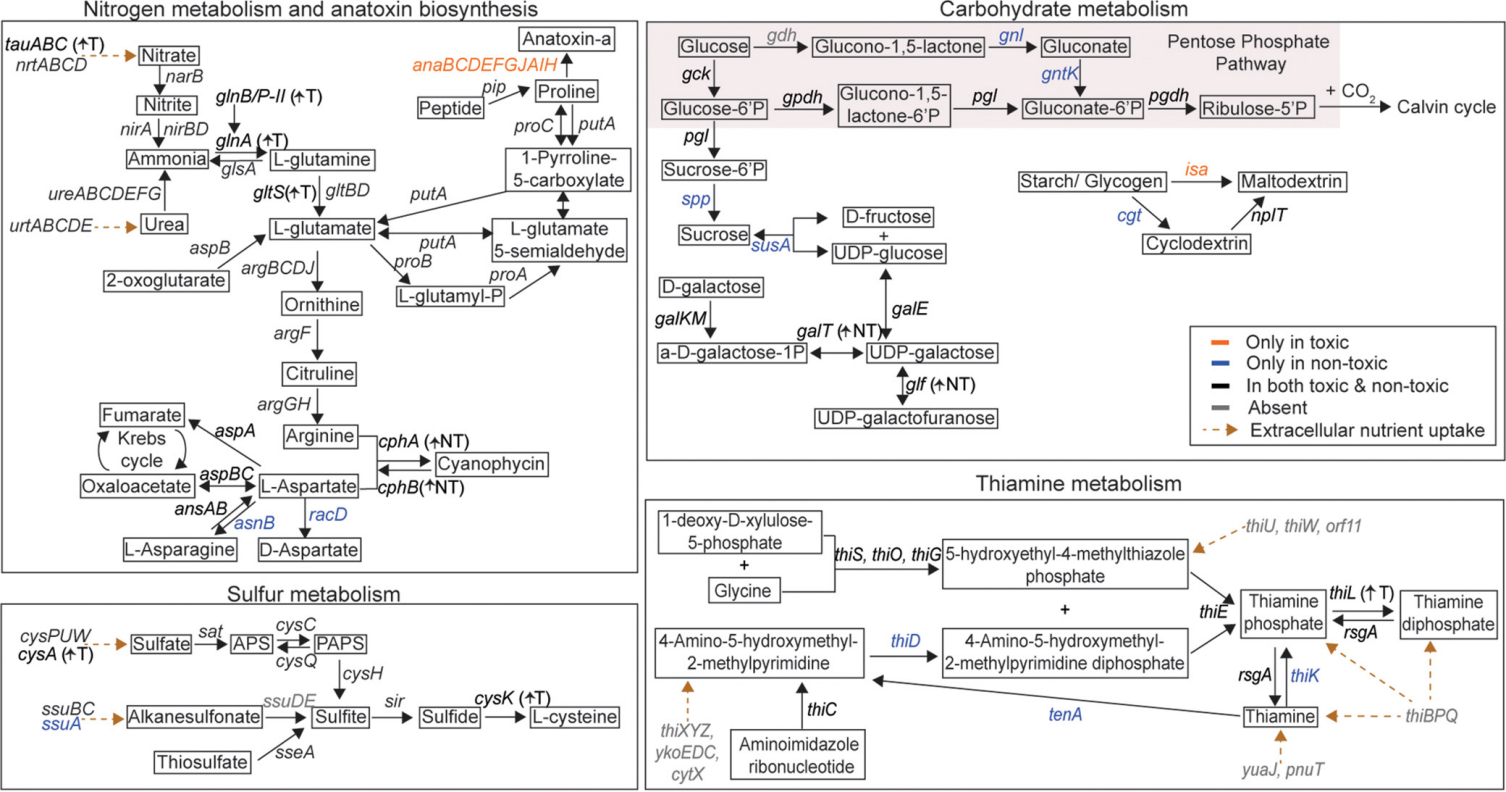
Schematic of metabolic pathways in *Microcoleus* spp. (gene details available in Tables S4 and S5). ↑NT represents more copies of genes in nontoxic strains, whereas ↑T depicts more copies of genes in toxic strains. Text color indicates the gene distribution across the *Microcoleus* genomes, as shown in the box on the right side of the diagram. Brown dotted arrows represent uptake of extracellular nutrients.

KEGG pathway analysis highlighted that, aside from BGCs, toxic and nontoxic strains utilize different carbon storage/breakdown, nutrient acquisition and transport, and chemosensory and stress adaptation mechanisms (additional details in Text S1, Fig. S4, and Table S4). Significantly more orthologs involved in energy metabolism, biofilm formation, motility, and cell growth and death were found in nontoxic strains (Benjamini-Hochberg adjusted *P* value < 0.05; Fig. 3). Results further predict that nontoxic strains can synthesize sucrose and thiamine and take up alkanesulfonate as an alternative sulfur source, while toxic strains cannot (Fig. 7). Instead, there were significantly more orthologs related to lipid metabolism (FabD and MCH) in toxic strains (Fig. 3 and Fig. S4), which are implicated in anatoxin biosynthesis. The biosynthesis of anatoxin-a starts with proline adenylation by AnaC and attachment to an acyl carrier protein, AnaD (also known as FabD) (64). AnaA acts as a thioesterase (also known as MCH) and is involved in the final hydrolysis step in anatoxin production (65).

10.1128/mBio.02235-21.4 FIG S4 Heatmap of significantly different KEGG gene clusters between toxic and nontoxic *Microcoleus* that are involved in key metabolism pathways. The *x* axis shows genes. The *y* axis shows genomes. Squares in the heatmap are assigned colors across a gradient from yellow (least) to blue (highest) based on the scaled number of gene copies found. Hierarchical clustering was done based on the ward.D2 algorithm. Text color indicates the nontoxic (grey), nontoxic clustered with toxic (orange), and toxic (blue) *Microcoleus* groups. Download FIG S4, PDF file, 0.6 MB. Copyright © 2021 Tee et al. 2021 Tee et al. https://creativecommons.org/licenses/by/4.0/ This content is distributed under the terms of the Creative Commons Attribution 4.0 International license.

10.1128/mBio.02235-21.9 TABLE S5 Genes that are present in all *Microcoleus* genomes. Download Table S5, XLSX file, 0.01 MB. Copyright © 2021 Tee et al. 2021 Tee et al. https://creativecommons.org/licenses/by/4.0/ This content is distributed under the terms of the Creative Commons Attribution 4.0 International license.

### Variations in thiamine and sucrose biosynthesis.

Thiamine (vitamin B_1_) is an essential coenzyme that catalyzes transformations of carbon and biosynthesis of branched-chain amino acids in all living systems (66). The active form of the vitamin is thiamine diphosphate (TPP). Reductive evolution of bacterial genomes may lead to multiple auxotrophies, including vitamins and amino acids, which lead to dependent relationships with coexisting organisms (67–69). Experiments indicate that thiamine produced by phytoplankton can be used by cocultured auxotrophs to sustain their growth without additional thiamine supply (70). We found a complete thiamine biosynthesis pathway (*tenA*, *rsgA*, *thiCDEGKLOS*) in the nontoxic strains, but not in toxic strains (Fig. 7 and Fig. S4). The loss of essential genes, *thiD*, *tenA*, and *thiK* for thiamine biosynthesis, salvage, and phosphorylation pathways in the reduced genomes of toxic *Microcoleus* may indicate thiamine auxotrophy and a dependency on cohabiting nontoxic strains to acquire vitamins. According to the Black Queen hypothesis, reductive evolution in free-living bacteria and associated gene loss can lead to dependencies on leaky “helper” organisms (71). Loss of function in toxic strains resulting in dependence on nontoxic strains for specific metabolites would explain why both toxic and nontoxic *Microcoleus* always co-occur in the natural environment (5, 11). We detected no thiamine uptake genes in any of the *Microcoleus* strains; however, this may be due to a lack of references. Thus far, no thiamine transporter system has been identified in cyanobacteria (72, 73). While no thiamine auxotrophy in cyanobacteria has been reported previously (72), many harmful algal bloom species, including some dinoflagellate and diatom species, are known thiamine auxotrophs (74, 75).

Orthogroups responsible for sucrose synthesis (sucrose-6-phosphatase [*spp*] and sucrose synthase [*susA*]) were only present in nontoxic strains (Fig. 7). Sucrose serves as one of the major compatible solutes among cyanobacteria in freshwater habitats, and the intracellular accumulation of sucrose helps cyanobacteria cope with salt stress (76–78). Missing genes related to sucrose synthesis suggest that toxic strains may be less tolerant to salt stress, possibly due to expendable gene loss, as they exclusively inhabit freshwater environments (12) and thus encounter minimum fluctuations in salinity.

### Differences in starch and glucose utilization.

Excess polysaccharide synthesized via photosynthesis during the day is stored as glycogen and starch in cyanobacteria (79). These storage molecules are then consumed to maintain levels of ATP and NADPH at night. Our data indicate that toxic and nontoxic *Microcoleus* utilize distinct enzymes for maltodextrin biosynthesis (Fig. 7 and Fig. S4)—a key intermediate product in glycogen and starch degradation. Toxic strains break down glycogen/starch into maltodextrin via isoamylase (ISA), whereas nontoxic strains first degrade starch into cyclodextrin via cyclodextrin glucosyltransferase (CGT), which is then converted into maltodextrin via cyclomaltodextrinase (NplT) (Fig. 7). Cyclodextrin, which aids in drug/compound delivery, has been reported to improve antifungal activity in *Anabaena* spp. (80), suggesting that nontoxic *Microcoleus* may potentially produce cyclodextrin to enhance the delivery of other antibacterial or antifungal compounds.

The pentose phosphate pathway (PPP) is a crucial mechanism for the oxidation of glucose and NADPH generation in most organisms (81). The product, ribulose-5-phosphate, can be phosphorylated into ribulose-1,5-diphosphate in the dark reaction-Calvin cycle. A complete PPP that generates ribulose-5-phosphate from glucose (*gck*) and then glucose-6-phosphate (*gpdh*, *pgl*, *pgdh*) was found within *Microcoleus* genomes (Fig. 7). Results indicate that nontoxic strains are also able to use an alternative nonphosphorylated route for directing the intermediate, gluconolactone, into the PPP, which bypasses the rate-limiting enzyme glucose 6-phosphate dehydrogenase (G6PDH) (82, 83). The genes involved transform gluconic-acid/gluconolactone to gluconate-6-phosphate (*gnl* and *gntk*), which can then be converted to ribulose-5-phosphate by *pgdh*. However, the glucose dehydrogenase gene, *gdh*, which is involved in the breakdown of glucose to gluconolactone, the first step of the nonphosphorylated route, is missing from *Microcoleus* genomes. This suggests that the nontoxic strains may possess novel enzymes that substitute for glucose dehydrogenase, or they can acquire and utilize gluconolactone from the environment.

### Distinct nitrogen, phosphorus, and sulfur acquisition and storage mechanisms in toxic and nontoxic groups.

Analysis of pathways for nitrogen metabolism showed toxic and nontoxic *Microcoleus* differ in their capacity to take up and store nitrogen. While all *Microcoleus* strains harbor the classic high-affinity nitrate/nitrite transport (Nrt) and urea uptake (Ure) systems, toxic strains also harbor an additional nitrate/sulfonate/taurine transport system (NitT/TauT) (Fig. 7). This suggests that toxic strains have a greater capacity to acquire nitrogen and proliferate in a nitrogen-rich environment, which corroborates prior findings that river sites with higher nitrogen concentrations tended to have higher relative abundances of toxic versus nontoxic strains (8). However, results here show that nontoxic species are likely to be more resistant to variations in nitrogen supply. An alternative pathway for nitrogen assimilation among *Microcoleus* species involves cyanophycin synthesis and catabolism, by CphAB (19), which functions as temporary nitrogen/carbon storage (84). Toxic *Microcoleus* strains are equipped with one cyanophycin gene cluster (Fig. 7). In contrast, nontoxic strains are equipped with two distinct cyanophycin gene clusters. The additional gene cluster may increase maximum rates of cyanophycin metabolism, helping nontoxic strains to adapt to fluctuating nitrogen concentrations in the environment (84).

Results indicate a link between nitrogen acquisition and toxin production. Glutamate is known to be the most common precursor for proline biosynthesis (85), which plays a significant role in anatoxin production. Besides acting as a building block for secondary metabolites, glutamate is essential for nitrogen assimilation (86). Although genes responsible for proline production are present in both toxic and nontoxic strains, more copies of glutamine synthetase, *glnA*, and glutamate synthase, *gltS*, were found in toxic strains than in nontoxic strains (Fig. 7). Toxic strains, therefore, may accumulate more glutamine/glutamate, contributing to anatoxin biosynthesis and greater nitrogen assimilation. In addition, *Microcoleus* mats frequently proliferate in low-phosphate water and are able acquire/uptake nutrients under nutrient-limiting conditions (8, 12, 19). Accordingly, *Microcoleus* genomes in the present study contain diverse phosphate acquisition mechanisms (Table S5). While all *Microcoleus* strains harbor at least one copy of phosphatase and phosphate transporter orthologs, some toxic strains harbor multiple copies (Fig. S4), which potentially results in more efficient phosphate acquisition than in nontoxic counterparts.

Evidence suggests toxic strains invest greater resources into cysteine production. All *Microcoleus* strains have the capacity to acquire sulfur via a complete assimilatory sulfate reduction pathway (Fig. 7). Extracellular sulfate is transported into the cells via the high-affinity sulfate/thiosulfate uptake system, CysPUWA, reduced to sulfide via sulfite reductase (SiR), and then incorporated into cysteine by cysteine synthases (CysK or CysM). CysK can also bind and activate antibacterial toxins upon entry into target cells (87). Multiple copies of the *cysK* cysteine synthase genes and *cysA* transporter ATP binding subunit were found in toxic strains, while on average only one copy was found in nontoxic strains, implying toxic strains utilize CysK to perform additional biological functions. Additional copies of *cysK* and *cysA* may also enhance cysteine uptake, as has been shown for other genes (e.g., additional cloned copies of threonine synthase and transporter genes were shown to increase threonine production [88]). Greater synthesis of cysteine by toxic *Microcoleus* may be needed to overcome attrition of cysteine-bearing proteins. Previous studies on hepatotoxin-microcystin have shown that microcystin can covalently bind to the cysteine residue of specific proteins, which interferes with the stability and activity of these cysteine-bearing proteins (89, 90).

### Conclusion.

This study highlights remarkable genomic and metabolic differences within the *Microcoleus* genus, leading to their divergent evolution. Results indicate toxic strains adopted a genome-streamlining strategy, resulting in smaller genomes, fewer BGCs, and smaller noncoding gene fractions. The estimated maximum growth rate for the toxic group is lower, suggesting that ecological trade-offs likely accompany anatoxin production in *Microcoleus*. Such trade-offs are further characterized by metabolic deficiencies, including sucrose and thiamine synthesis genes, and other stress response mechanisms. Toxic and nontoxic strains may employ both cooperative strategies (by offering protection and sharing vitamins) and competitive strategies (nutrient uptake and assimilation) to coexist in mats and utilize the same resource pool. Genomic evidence leads us to predict that nontoxic *Microcoleus* strains synthesize and share thiamine with cohabiting toxic *Microcoleus*, while anatoxin-producing strains, by triggering PCD and releasing intracellular anatoxin, provide a resource to coexisting nontoxic strains (potentially affording protection from predation, oxidative stress, or nutrient limitation). Toxic and nontoxic strains are differently equipped with additional sets of nitrate transporter and cyanophycin genes, which likely confer distinct competitive advantages under fluctuating nitrogen availability. Understanding the genomic features that differentiate nontoxic versus toxic groups, provides a basis for assessing how different environmental factors affect their selection and proliferation in freshwater systems.

## MATERIALS AND METHODS

### Cyanobacterial strains and genome sequencing.

The genomic features and metadata of 42 *Microcoleus* strains, including 28 MAGs and 14 isolates, used in this study are shown in Table S1. Representatives were selected for this study based on close similarity to *Microcoleus autumnalis* (8, 19, 30) and originated from 2 countries and 13 rivers. Of these, 11 nonaxenic *Microcoleus* cultures were obtained from the Cawthron Institute’s Culture Collection of Micro-algae (CICCM, Nelson, New Zealand; http://cultures.cawthron.org.nz). These strains were grown in liquid MLA medium (91) and incubated under standard conditions (160 ± 20 mmol photons m^−2^ s^−1^; 12:12 h light:dark cycles; 18 ± 1°C) for at least 3 weeks to achieve adequate biomass for DNA extraction. DNA was extracted from 0.1 to 0.4 g of each sample using a DNeasy PowerSoil DNA kit (Qiagen, USA). Genomic libraries with 550-bp insert sizes were prepared with a TruSeq DNA nano library preparation kit, and 2 × 250-bp sequencing was performed using the Illumina HiSeq 2500 platform with V2 chemistry at the Otago Genomics Facility (University of Otago, New Zealand). The Illumina sequence reads of three additional nonaxenic *Microcoleus* cultures from the United States (30) were downloaded from the NCBI database (92). The 28 environmental metagenome-assembled *Microcoleus* genomes and their corresponding Illumina sequence reads were obtained from prior studies (8, 19).

### Read processing and genome assembly.

All sequence reads associated with the 42 strains were quality checked using FastQC (93). Adapter sequences were removed, and reads were trimmed with bbduk.sh from BBMap v37.93 (94); only those with a quality score of ≥30 and length of ≥80 bp were retained. Trimmed reads from each CICCM-derived culture, and the additional three nonaxenic *Microcoleus* isolates from the United States, were assembled using metaSPAdes (95) with k-mer values of 41, 61, 81, 101, and 127. Scaffolds larger than 2 kb were binned using MetaBAT (96), CONCOCT (97), and MaxBin 2.0 (98). The highest-quality nonredundant prokaryotic bins from each assembly were selected using DASTool (99) and CheckM v1.0.13 (100) and were used to estimate genome completeness and contamination. All bins (11 in total) were manually curated and validated using VizBin (101). Genome coverage was calculated by mapping reads to genomes using Bowtie v1.2.0 (102), allowing ≤1 mismatch per read pair, showing that >10 times read coverage was obtained for each bin (Table S1). Estimated genome size was calculated as follows: total number of bases in the genome × 100/CheckM-estimated completeness based on lineage-specific marker genes.

### Small subunit (SSU) rRNA gene reconstruction.

Full-length 16S rRNA sequences were reconstructed from the trimmed reads of all 42 strains, over 50 iterations, using EMIRGE (103) with the SILVA SSURef NR99 132 database (104) and a clustering threshold of 99%.

### Genome annotation and prediction of minimum generation time.

The 42 *Microcoleus* genomes were annotated as follows. Open reading frames (ORFs) were predicted using Prodigal (105) and subjected to orthologous protein grouping using OrthoFinder v2.3.3 (106) with default parameters. The orthologous clusters were classified as core, accessory, or unique according to their distribution across the genomes. The core orthogroups comprise predicted proteins shared by all strains; the accessory clusters incorporate proteins assigned to a subset of study strains; and the unique clusters include proteins assigned to only a single strain. The orthologous groups were then annotated using KofamKOALA and the Kyoto Encyclopedia of Genes and Genomes (KEGG) release 94.0 database (Table S4) (107). Genome sequences were screened for biosynthetic gene clusters (BGCs) using antiSMASH v4.2 (108), insertion elements were predicted and classified with ISEScan v1.7.1 (109), and genomic islands were predicted using Alien Hunter v1.7.1 (110). Anatoxin gene clusters and neighboring genes were aligned using Mauve multiple alignments (111). Snippy v4.4.0 (112) was used for variant calling (SNPs or insertion/deletion) and generation of core genome SNP alignments among all *Microcoleus* genomes. Minimum generation times were estimated for all *Microcoleus* genomes based on the codon usage bias from a set of typically highly expressed genes (mainly rRNA, tRNA) using growthpred (113) with the parameters “-b -c 0 -r –T 20 -S.”

### Whole-genome comparisons and phylogenetic analyses.

Whole-genome comparisons were conducted via BLASTN and visualized using the BLAST Ring Image Generator (BRIG [114]). Genome sequences related to *Microcoleus* were retrieved from the NCBI genome database (92) (Table S1). The phylogenetic relationship of *Microcoleus* genomes was inferred by pairwise nucleotide-level comparisons based on digital DNA-DNA hybridization (dDDH) (41, 115) and average nucleotide identity (ANI) (116) values. Pairwise dDDH values were estimated using the Genome-To-Genome Distance Calculator (GGDC) v2.1 with a threshold of 70% for species delineation, which corresponds to at least 96.5% ANI and an alignable gene fraction of >70% (42). Reciprocal BLASTN was carried out for all genomes to calculate ANI. Maximum-likelihood trees with branch supports were constructed based on reconstructed 16S rRNA genes, 12,421 core single nucleotide polymorphisms (SNPs), and concatenated alignments of 120 single-copy core marker genes obtained from GTDB-Tk v0.2.1 (117). Trees were built using the ultrafast bootstrap approximation (118) in IQ-TREE v1.6.9 (119). The species phylogenetic tree was inferred based on 525 single-copy core orthologs (347,012 amino acid) using FastTree v2.1.10 (120).

### Cyanotoxin measurement.

Anatoxin-a, homoanatoxin-a, and structural variants were measured using LC-MS/MS, as described in a previous study (3). Biofilm samples were lyophilized and resuspended in 10 ml of Milli-Q water with 0.1% formic acid. Samples were then separated by liquid chromatography (Waters Acquity UPLC; Waters Corp., Massachusetts, USA) on a BEH C_18_ column (1.7 μm, 1 × 50 mm; Waters Corp.) and quantified on a Quattro Premier XE triple quadrupole mass spectrometer (Waters-Micromass, Manchester, UK).

### Statistical analysis.

The following statistical analyses were carried out in R environment version 4.0.2 (121). A Wilcoxon rank-sum test was performed to determine the significant differences of genome sizes, GC content, predicted maximum growth rate, number of insertion sequences, number of biosynthetic gene clusters, and orthogroup count between toxic and nontoxic *Microcoleus* strains. Pearson correlation coefficients and regression lines between genome size and the gene content and estimated genome completeness were calculated and plotted using the stat_cor function in ggpubr (122) and geom_smooth function in the ggplot2 package (123). Heatmaps with ward.D2 hierarchical clustering were plotted using pheatmap (124).

### Data availability.

The data generated in this study are publicly available. All sequence data have been deposited with NCBI under BioProject PRJNA733706. Codes for read processing and genome assembly are available at https://github.com/HweeSze/Microcoleus_comparative_manuscript.
