# Supplementary material for: Genome Streamlining, Plasticity, and Metabolic Versatility Distinguish Co-occurring Toxic and Nontoxic Cyanobacterial Strains of Microcoleus
Source: mBio. 2021 Oct 26;12(5):e02235-21. doi: 10.1128/mBio.02235-21 (PMC8546630; doi:10.1128/mBio.02235-21)
Supplement: TEXT S1 [file mbio.02235-21-s0001.docx]

**Supplementary Result**

*Additional gene clusters related to chemosensory and stress adaptation in non-toxic Microcoleus*

Both toxic and non-toxic *Microcoleus* possess multiple genes related to the chemosensory pathway, *cheABCWR*, for cell regulation, motility, and biofilm formation, to support growth in diverse habitats(1, 2) (Table S5). However, non-toxic *Microcoleus* harbour an additional set of chemotaxis genes, *wspBCADEF* (Fig. S4)*,* similar to the *wsp* gene cluster previously reported in *Phormidesmis priestleyi* (3). This gene cluster encodes a chemoreceptor *wspA* and an adenylate/guanylate cyclase domain-containing response regulator (which generates cAMP/cGMP signalling molecules) (4). Mat formation is known to be triggered by cAMP in *Spirulina* sp. (5), whereas cGMP has been reported to mediate signal transduction and adaptation to UV-B stress (6), suggesting the *wsp* gene cluster and the associated cyclase domain-containing response regulator may promote mat proliferation or act as an additional stress response mechanism in non-toxic strains. In addition to chemosensory pathways, *Microcoleus* has adopted multiple stress- and immunity-related mechanisms for adaptation to diverse ecological niches, including UV-induced DNA excision repair genes *uvrABCD*, osmoregulatory genes (*opuABCD*, *trkAH*, *kdpABC*, *cvrA,* and *nhaP2*), and genes encoding heat-shock molecular chaperones (*dnaJK* and *groESL,* Table S5 and Fig. S4). The majority of stress-related orthogroups were significantly more prevalent in non-toxic strains (Fig. S4), reflecting a greater capacity to acclimate and prevail under different environmental stresses (heat, light, cold, desiccation, and oxidative stress) (7, 8).

**References**

1. He K, Bauer CE. 2014. Chemosensory signaling systems that control bacterial survival. Trends Microbiol 22:389–398.

2. Huang Z, Wang Y-H, Zhu H-Z, Andrianova EP, Jiang C-Y, Li D, Ma L, Feng J, Liu Z-P, Xiang H, Zhulin IB, Liu S-J. 2019. Cross talk between chemosensory pathways that modulate chemotaxis and biofilm formation. mBio 10:e02876-18.

3. Chrismas NAM, Barker G, Anesio AM, Sánchez-Baracaldo P. 2016. Genomic mechanisms for cold tolerance and production of exopolysaccharides in the Arctic cyanobacterium *Phormidesmis priestleyi* BC1401. BMC Genomics 17:533.

4. Biswas KH, Shenoy AR, Dutta A, Visweswariah SS. 2009. The evolution of guanylyl cyclases as multidomain proteins: conserved features of kinase-cyclase domain fusions. J Mol Evol 68:587–602.

5. Ohmori M, Okamoto S. 2004. Photoresponsive cAMP signal transduction in cyanobacteria. Photochem Photobiol Sci 3:503–511.

6. Cadoret J-C, Rousseau B, Perewoska I, Sicora C, Cheregi O, Vass I, Houmard J. 2005. Cyclic nucleotides, the photosynthetic apparatus and response to a UV-B stress in the cyanobacterium *Synechocystis*  sp. PCC 6803. J Biol Chem 280:33935–33944.

7. Singh H. 2018. Desiccation and radiation stress tolerance in cyanobacteria. J Basic Microbiol 58:813–826.

8. Webb R, Sherman LA. 1994. The cyanobacterial heat-shock response and the molecular chaperones, p. 751–767. *In* Bryant, DA (ed.), The Molecular Biology of Cyanobacteria. Springer, Dordrecht, Netherlands.
