# Supplementary figures and images for: Genome Streamlining, Plasticity, and Metabolic Versatility Distinguish Co-occurring Toxic and Nontoxic Cyanobacterial Strains of Microcoleus
Source: mBio. 2021 Oct 26;12(5):e02235-21. doi: 10.1128/mBio.02235-21 (PMC8546630; doi:10.1128/mBio.02235-21)

Tree scale: 0.1

### Microcoleus group

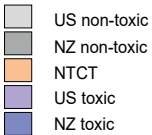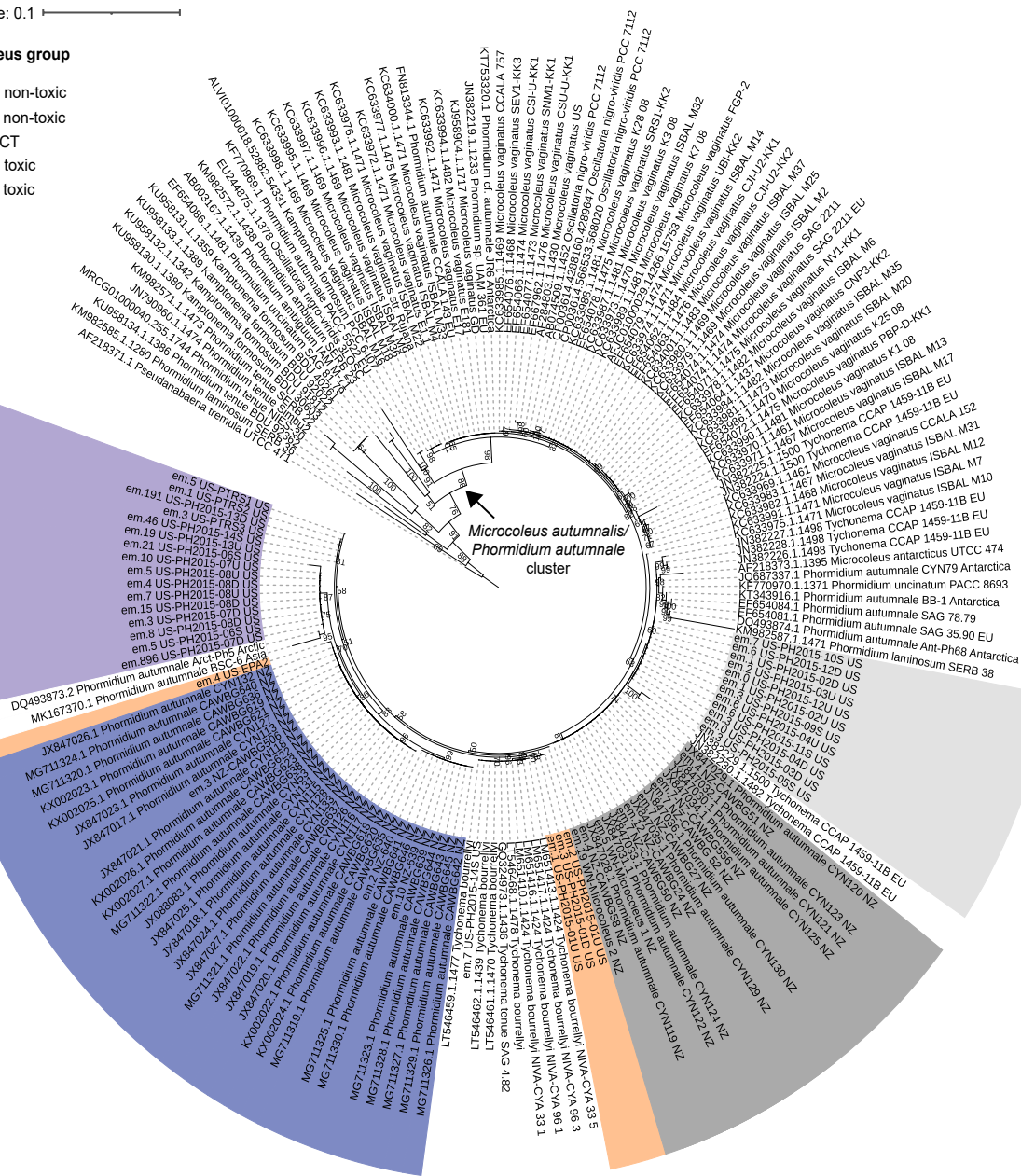

Supplement: FIG S1 [file mbio.02235-21-sf001.pdf]

60 70 80 90 100  
ANI value (%)

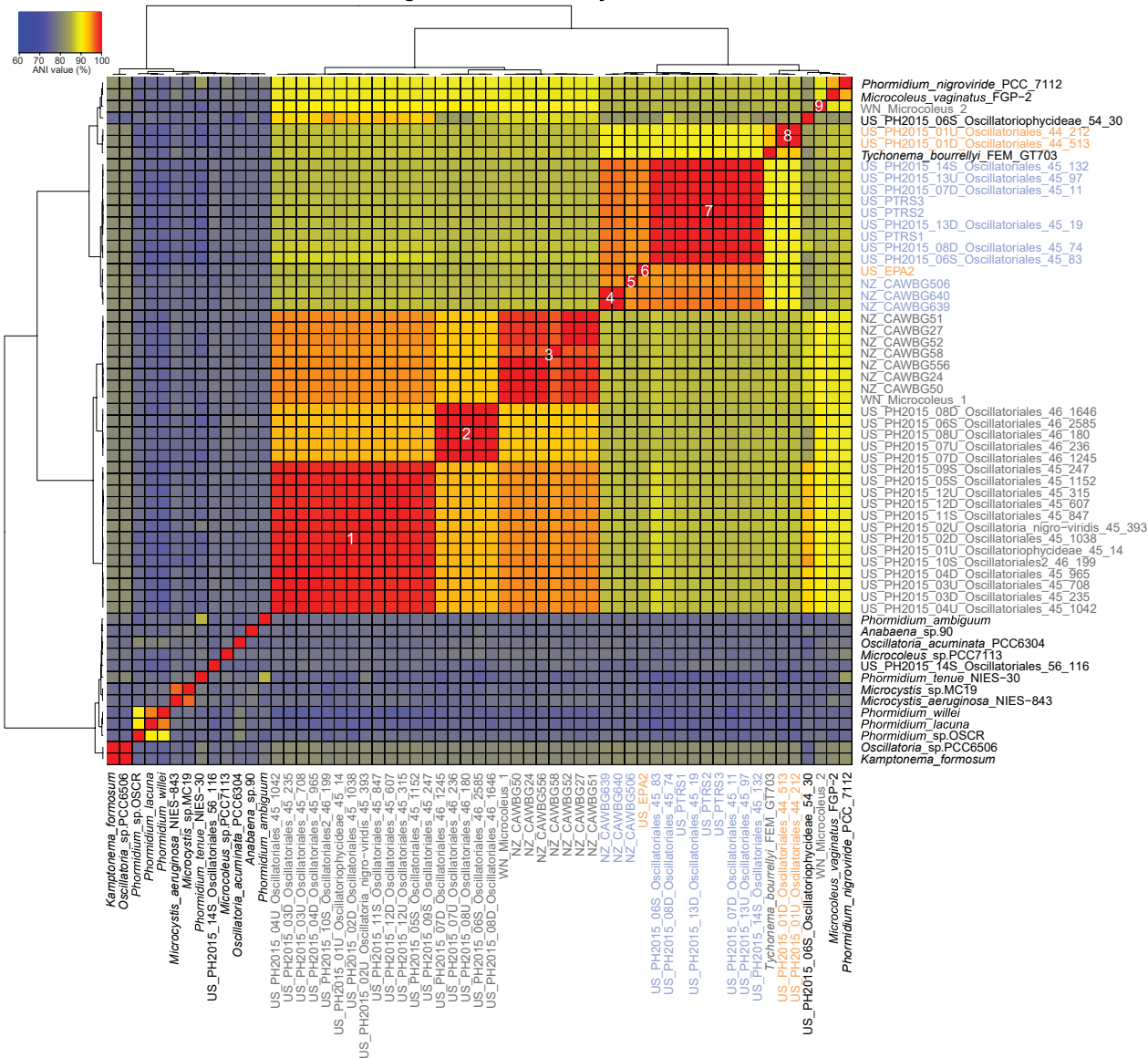

Supplement: FIG S3 [file mbio.02235-21-sf003.pdf]
