# Supplementary material for: Genome Streamlining, Plasticity, and Metabolic Versatility Distinguish Co-occurring Toxic and Nontoxic Cyanobacterial Strains of Microcoleus
Source: mBio. 2021 Oct 26;12(5):e02235-21. doi: 10.1128/mBio.02235-21 (PMC8546630; doi:10.1128/mBio.02235-21)
Supplement: FIG S4 [file mbio.02235-21-sf004.pdf]

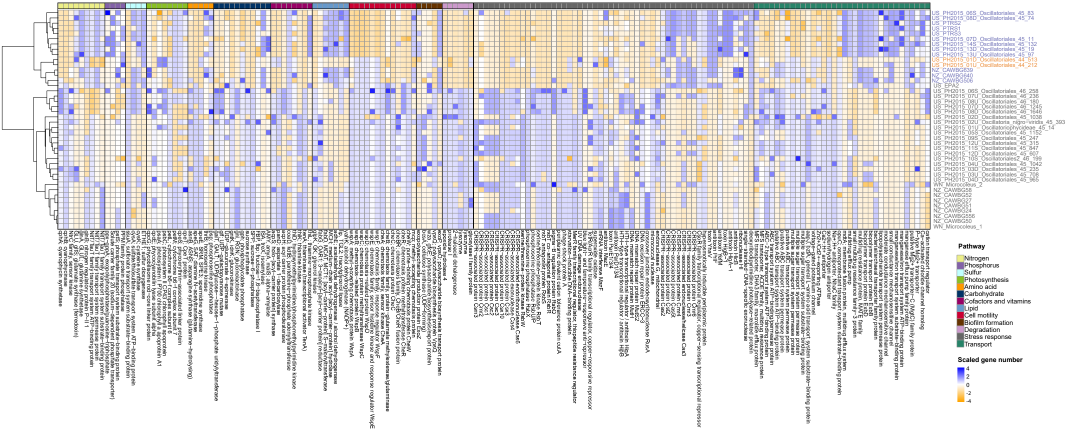
US_PH2015_06S_Oscillatoriales_45_83
US_PH2015_08D_Oscillatoriales_45_74
US_PTRS2
US_PTRS1
US_PTRS3
US_PH2015_07D_Oscillatoriales_45_11
US_PH2015_14S_Oscillatoriales_45_132
US_PH2015_13D_Oscillatoriales_45_19
US_PH2015_13U_Oscillatoriales_45_97
US_PH2015_01D_Oscillatoriales_44_513
US_PH2015_01U_Oscillatoriales_44_212
NZ_CAWBG639
NZ_CAWBG640
NZ_CAWBG506
US_EPA2
US_PH2015_06S_Oscillatoriales_46_258
US_PH2015_07U_Oscillatoriales_46_236
US_PH2015_08U_Oscillatoriales_46_180
US_PH2015_01S_Oscillatoriales_46_1245
US_PH2015_08D_Oscillatoriales_46_1646
US_PH2015_02D_Oscillatoriales_45_1038
US_PH2015_02U_Oscillatoria_nigro-viridis_45_393
US_PH2015_01S_Oscillatoriophycideae_45_14
US_PH2015_05S_Oscillatoriales_45_1152
US_PH2015_09S_Oscillatoriales_45_247
US_PH2015_12U_Oscillatoriales_45_315
US_PH2015_11S_Oscillatoriales_45_847
US_PH2015_12D_Oscillatoriales_45_607
US_PH2015_10S_Oscillatoriales2_46_199
US_PH2015_04U_Oscillatoriales_45_1042
US_PH2015_03D_Oscillatoriales_45_235
US_PH2015_03U_Oscillatoriales_45_708
US_PH2015_04D_Oscillatoriales_45_965
WN_Microcoleus_2
NZ_CAWBG58
NZ_CAWBG52
NZ_CAWBG27
NZ_CAWBG51
NZ_CAWBG24
NZ_CAWBG556
NZ_CAWBG50
WN_Microcoleus_1
Pathway
Nitrogen
Phosphorus
Sulfur
Photosynthesis
Amino acid
Carbohydrate
Cofactors and vitamins
Lipid
Cell motility
Biofilm formation
Degradation
Stress response
Transport
Scaled gene number
4
2
0
-2
-4
